# Supplementary material for: Structural mechanism for inhibition of PP2A-B56α and oncogenicity by CIP2A
Source: Nat Commun. 2023 Feb 28;14:1143. doi: 10.1038/s41467-023-36693-9 (PMC9974998; doi:10.1038/s41467-023-36693-9)
Supplement: Supplementary file 1 — Supplementary Information [file 41467_2023_36693_MOESM1_ESM.pdf]

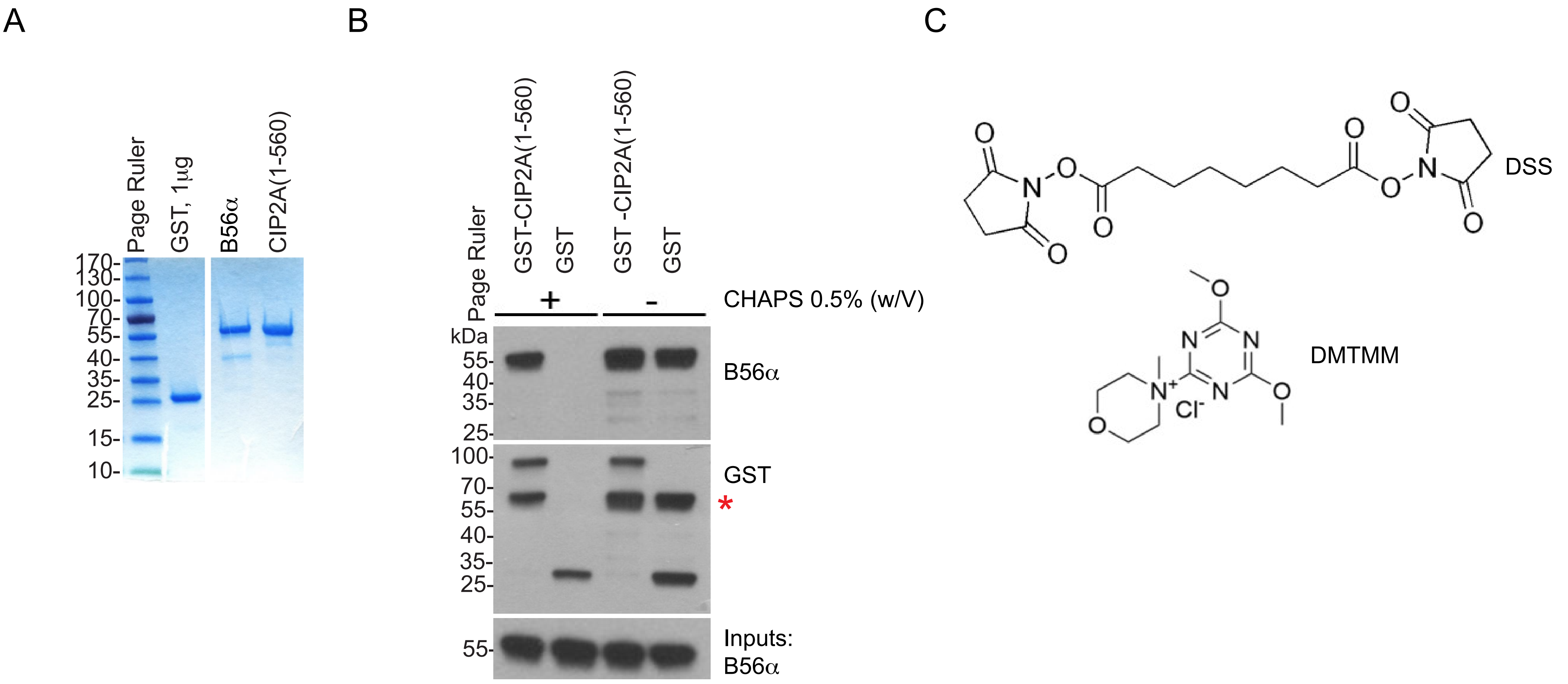

Supplementary Figure 1: CIP2A and B56α interaction is preserved under XL-MS suitable conditions. (A) Coomassie staining of the proteins used in XL-MS experiments. Coomassie stain of the protein preparations used in the binding assay was done once. (B) B56α and CIP2A were used in in vitro binding assay with 10 pmol purified recombinant protein, incubated with or without CHAPS detergent in the reaction buffer. The complexes were analyzed using Western blotting. Reaction was done in N=2 biological repeats. Red star indicates B56a signal from previous blotting. Source data are provided as a Source Data file. (C) Chemical structures of DSS (disuccinmidyl suberate) and DMTMM (4-(4,6-dimethoxy-1,3,5-triazin-2-yl)-4-methyl-morpholinium chloride).

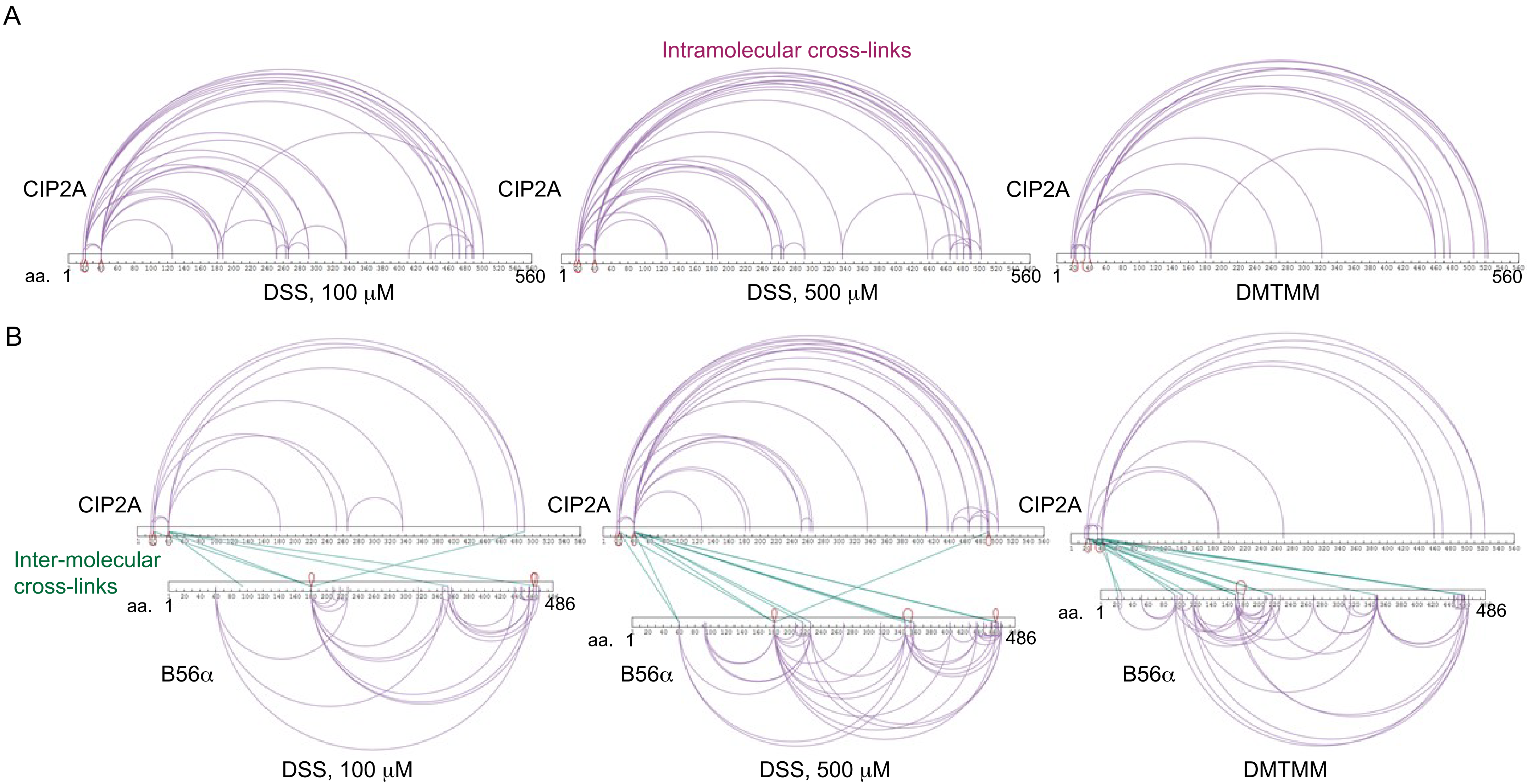

Supplementary Figure 2: Visualization of intra- and inter-molecular cross-linking on CIP2A)1-560) and B56α. (A) Data visualization for intra-molecular cross-links with CIP2A alone (purple lanes) using DSS, at 100 and 500 μM, and DMTMM. Increasing cross-linker concentration does not induce novel cross-links in the regions where they would not be present when using lower concentration of the cross-linking reagent. (B) Data visualization for CIP2A-B56α complex, with the same linker conditions like in A. Presence of B56α reduces conformational flexibility of CIP2A, as visible from fewer intra-molecular CIP2A contacts. In both panels, purple lines represent intra-molecular cross-links, green lines are inter-molecular cross-links, and in red are residues (lysine or glutamate) directly involved in the cross-links.

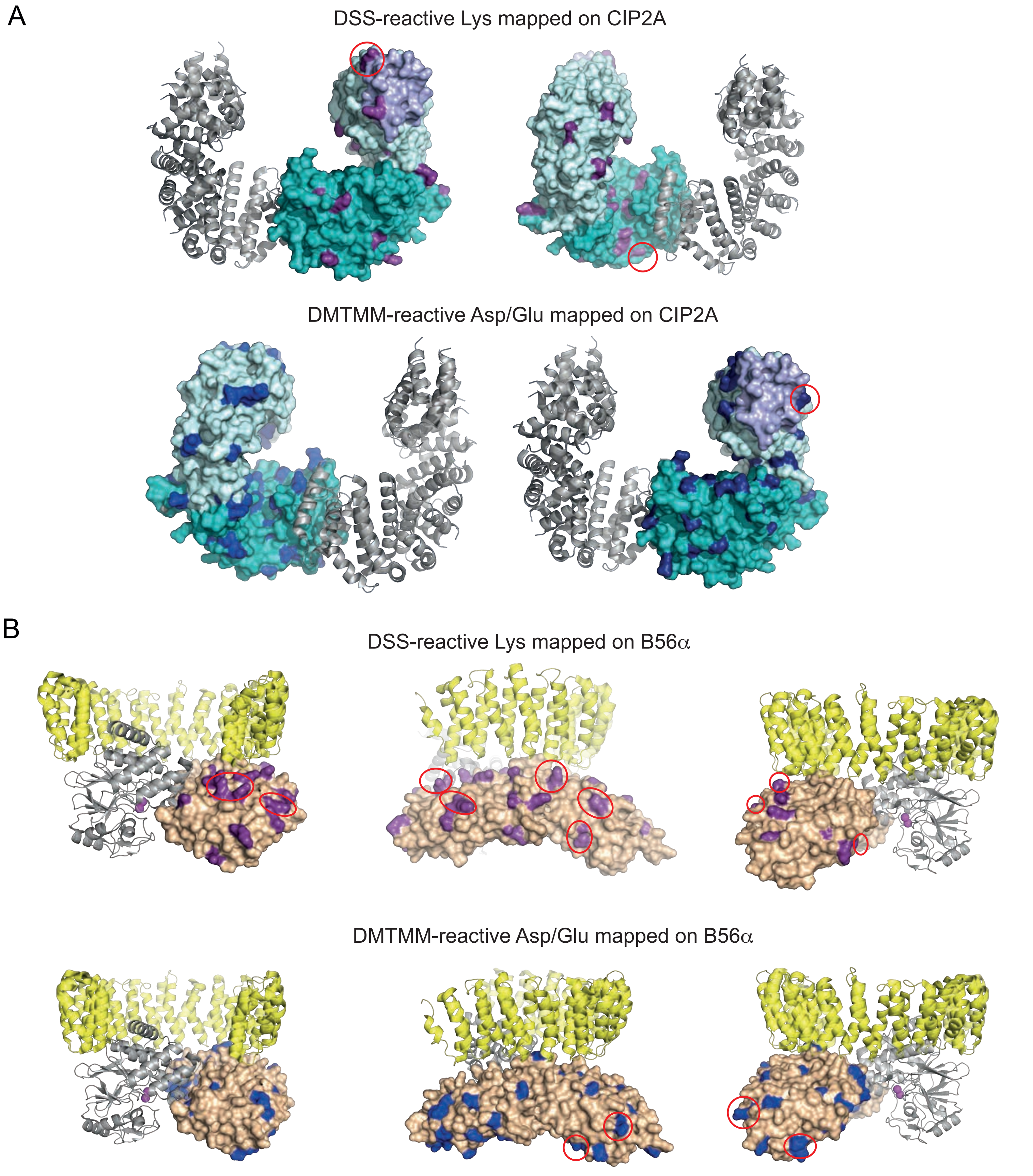

Supplementary Figure 3: Mapping distribution of amino acids reactive against cross-linking reagents, DSS and DMTMM, on the surface of CIP2A and B56. (A) CIP2A monomers are shown in green and blue. Lys are mapped in purple and Asp/ Glu in blue. (B) Representation for B56, with the same coloring legend as in (A). Red circles indicate aminoacids identified in actual CIP2A-B56 cross-links by XL-MS.

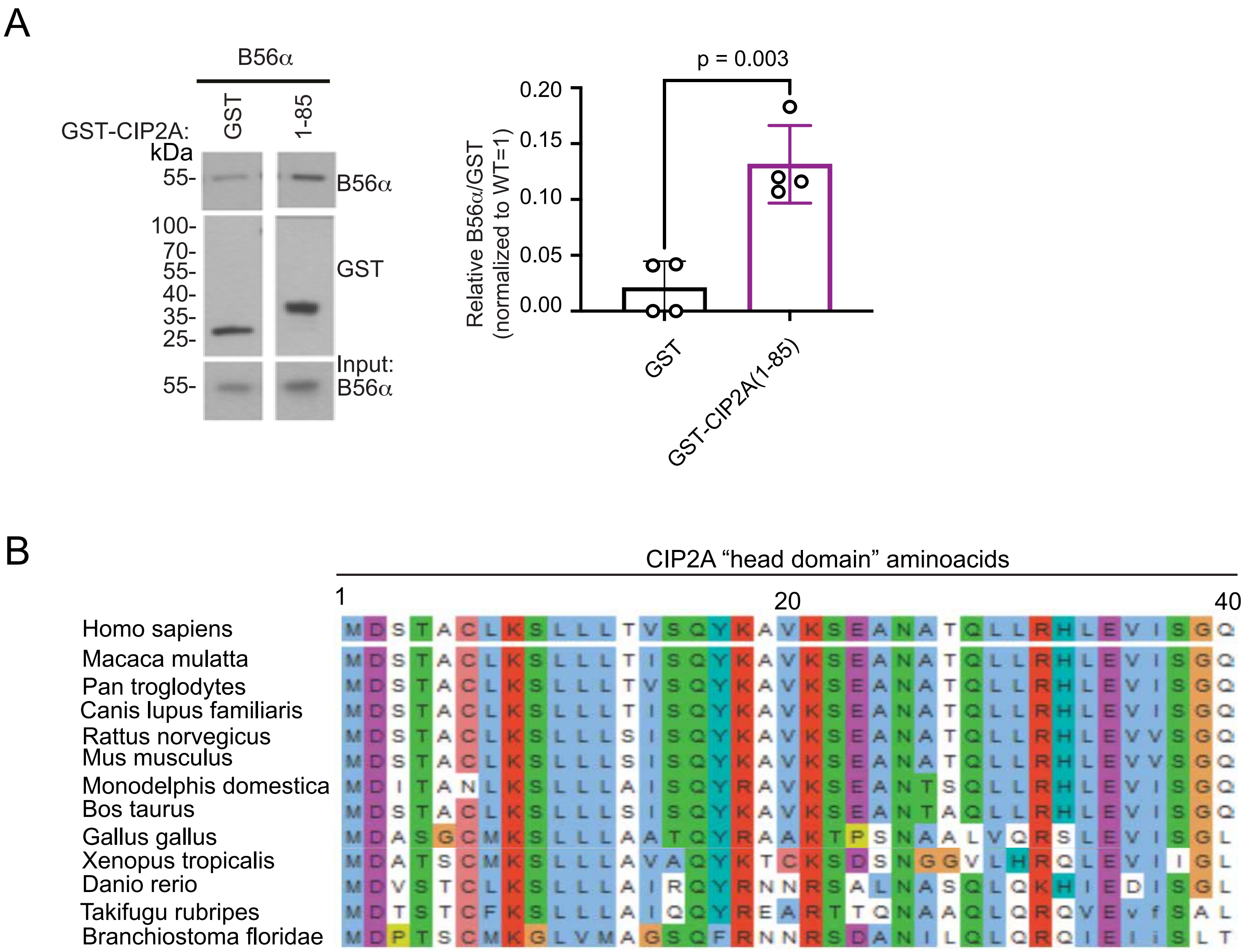

Supplementary Figure 4: Head domain of CIP2A interacts with PP2A-B56 and is conserved across species.

(A) GST-pulldown experiment demonstating direct interaction between the N-terminal CIP2A(1-85) fragment and B56α. Representative image from N=4 experiments is shown. Quantification shows mean + S.E.M. from N=4 biological repeats and was done using GraphPad Prism. Two-sided t-test; ** $p < 0.01$. Source data are provided as a Source Data file.

(B) Sequence conservation of CIP2A head domain across species.

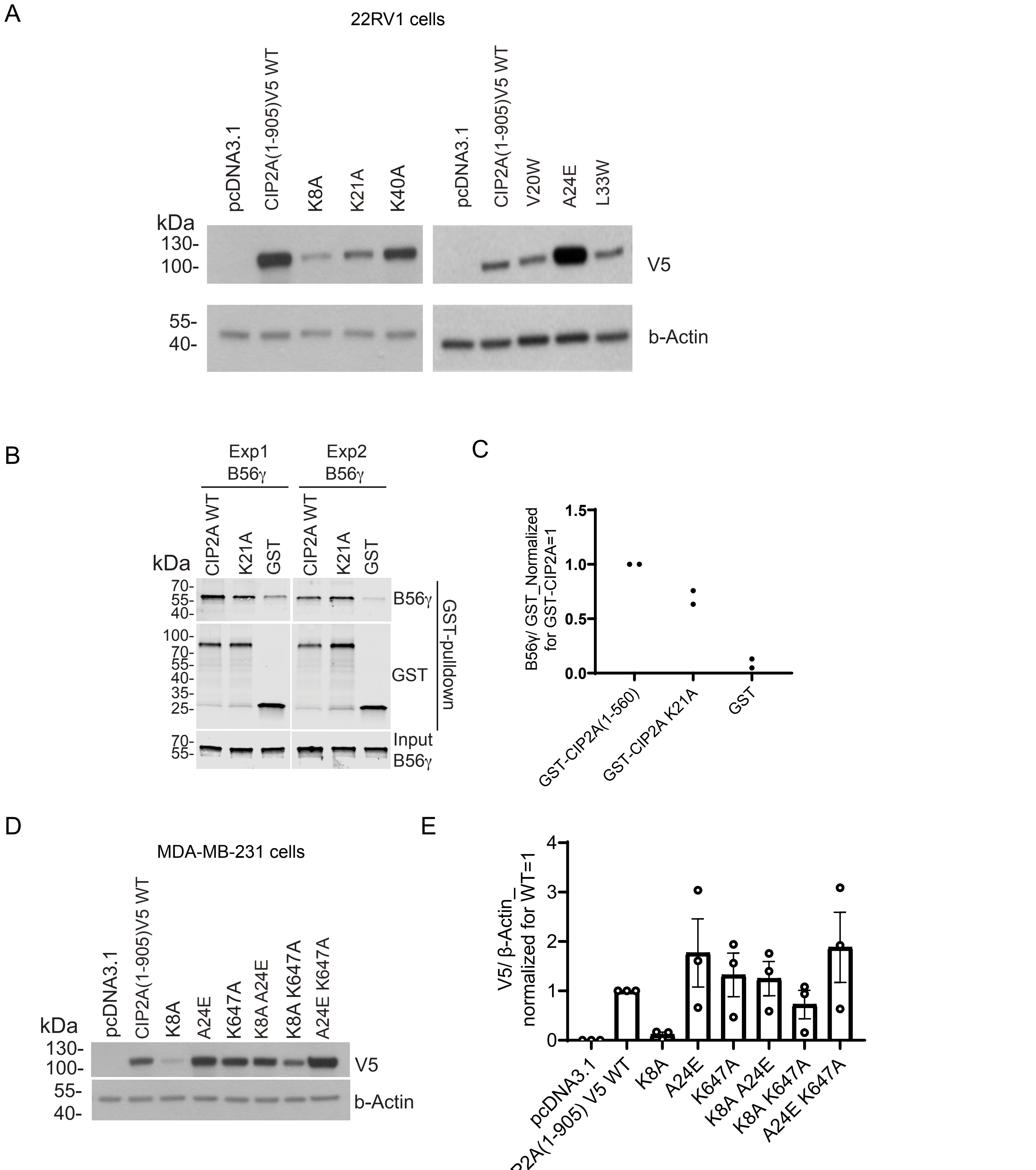

Supplementary Figure 5: Head domain of CIP2A modulate CIP2A protein expression in cancer cells. (A) Mutations in the N-terminal head domain of CIP2A modulate its protein stability in cell. Representative blots for the data shown in Fig. 2B are shown. (B) GST pulldown assay wuth CIP2A 1-560 WT (WT) or K21A mutant (K21A) with recombinant B56γ protein. (C) Quantification for panel B. B56γ signal was normalized to corresponding GST signal from the same pulldown sample. (D) K647A or A24E mutations rescues destabilizing effect of K8A mutation. Representative image from n=3 experiments is shown. (E) Quantification for panel D. Shown is mean + S.E.M. (A-E) Source data are provided as a Source Data file

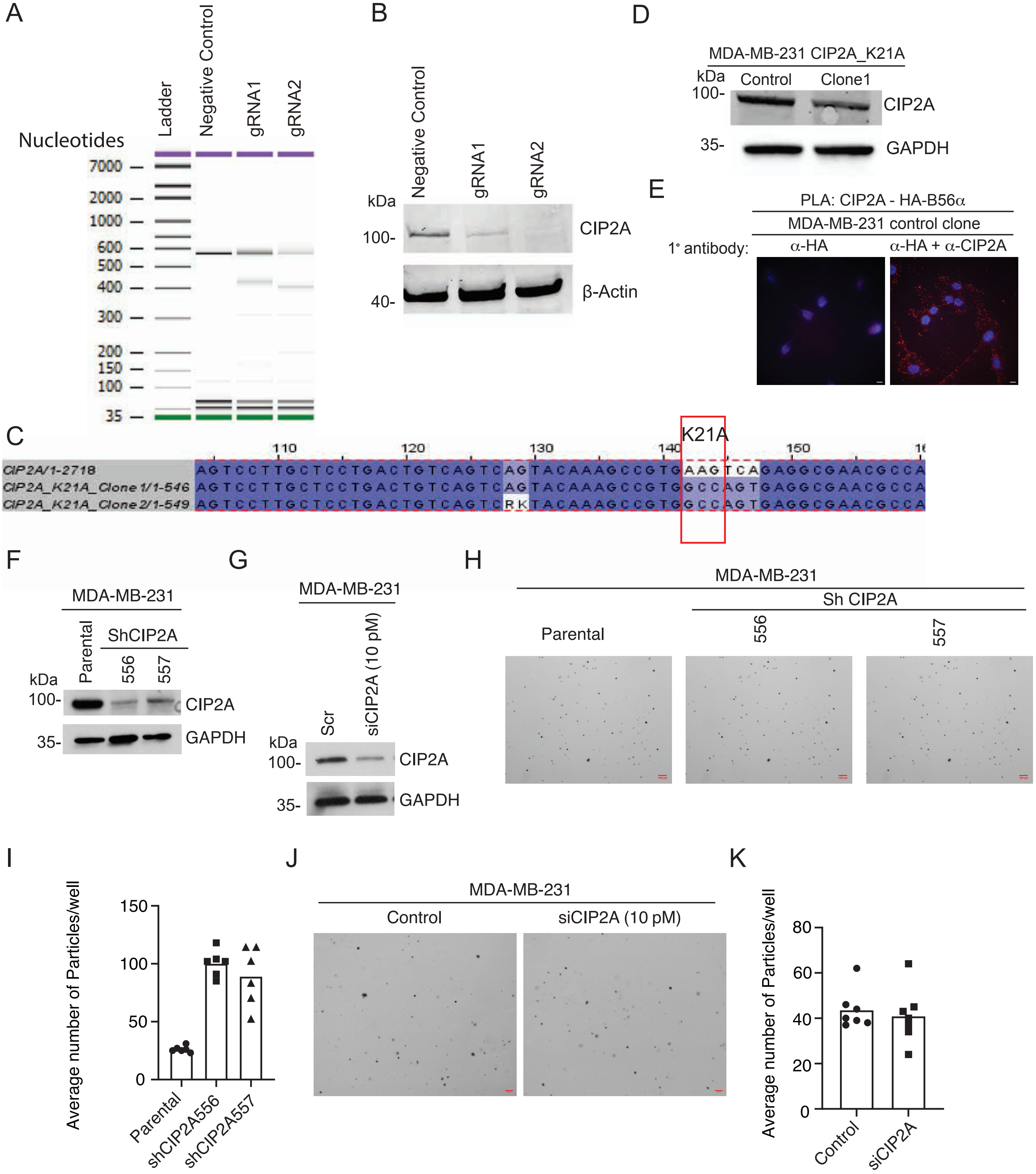

Supplementary Figure 6: CRISPR/Cas9-medidated knock-in of CIP2A (K21A) in MDA-MB-231. (A) Gel Image of T17E endonuclease digested fragment analyzed in Bioanalyzer to verify the genome targeting efficiency in the cell pools with two gRNAs as compared to negative control. (B) Western blot analysis of CIP2A expression (72 H) between negative control and two indepen dent gRNAs. N=1. C) Sanger sequencing analysis of two independent clonal cell lines of CIP2A_K21A (Clone 1 and Clone 2) compared to CIP2A WT, analyzed by NCBI Clustal W and displayed in Jar view. R and K letters in clone 2 sequence indicate undefined nucleotides. (D) Representative Western blot of CIP2A expression in control or K21A mutant MDA-MB-231 Crispr clones. (N=5). (E) Specificity control proximity ligation assay for interaction between HA-B56α and endogenous CIP2A. MDA-MB-231-Control cells were mock transfected and subjected to PLA including only primary antibody, either HA or HA and CIP2A. Red dot indicates positive PLA signal. Shown is a representative image from N=3. Scale bar 10uM. (F-G) Western blot analysis for panels H and J, respectively. (H) The effect of CIP2A knock-down on anchorage-independent growth in soft agar assays in MDA-MB-231 cells. The parental control or CIP2A shRNA-(sh556 and sh557) transduced MDA-MB-231 cells were subjected to anchorage-independent growth analysis (14 days of growth) by the soft agar assay. Representative image, scale bar = 100 μM. (I) Quantification for panel H, mean +/- SD from two independent biological replicates. (J) The effect of CIP2A depletion investigated by using siRNA (10 pmol). Representative image, scale bar = 100 μM. (K) Quantification for panel J. Shown is average number of particles per well from three technical replicates. (B,D,F,G,I, and K) Source data are provided as a Source Data file

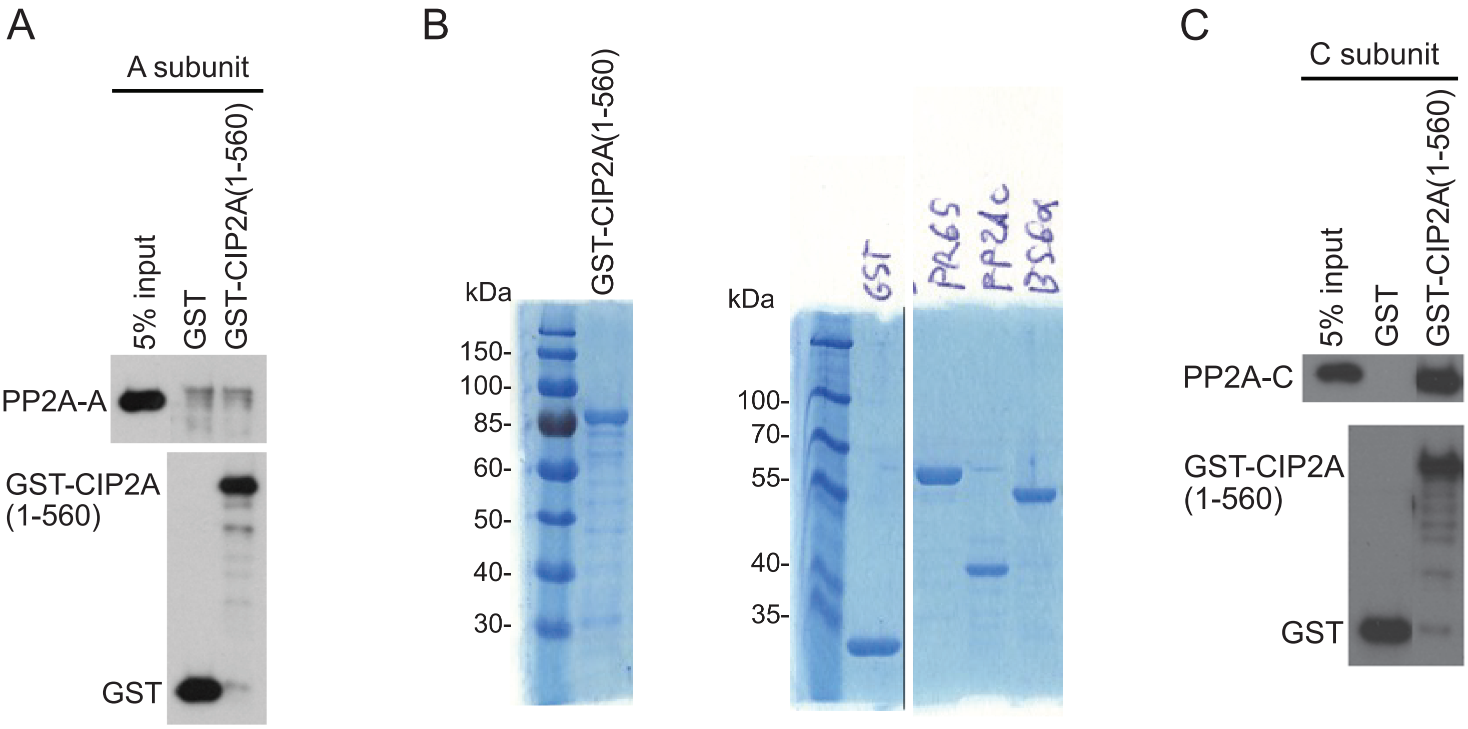

Supplementary Figure 7: CIP2A directly interacts with the catalytic PP2Ac subunit.
(A) GST pull-down assay comparing direct PP2A-A (A) subunit interaction between recombinant GST only or GST-CIP2A(1-560).
(B) Coomassie staining of indicated bacterially produced recombinant proteins PP2A-A (PR65), PP2A-C, and B56α.
(C) GST pull-down assay comparing direct PP2Ac interaction between recombinant GST only or GST-CIP2A(1-560). N=3 biological repeats (shown in the source data file). (A and C): Molecular size markers unavailable.

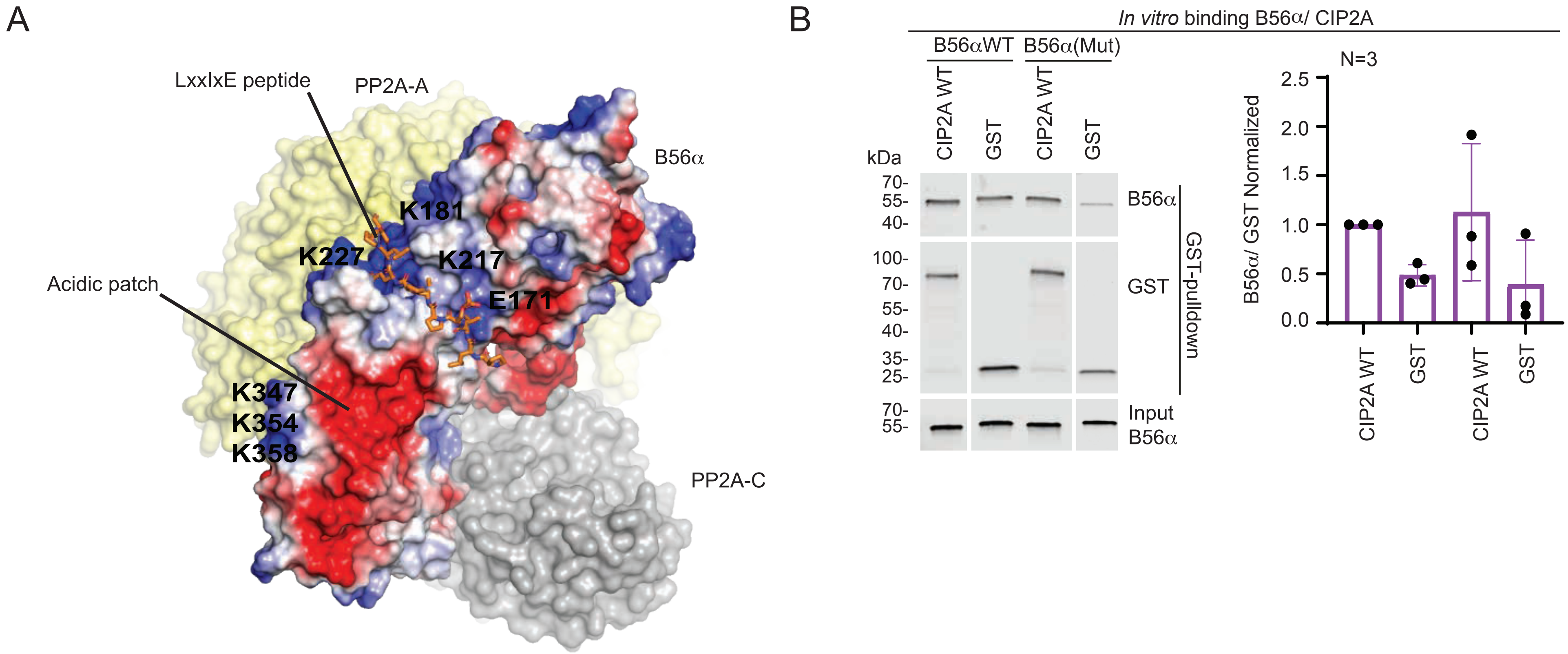
A
LxxIxE peptide
PP2A-A
B56α
K181
K227
K217
E171
Acidic patch
K347
K354
K358
PP2A-C
B
In vitro binding B56α/ CIP2A
B56αWT
B56α(Mut)
CIP2A WT
GST
CIP2A WT
GST
kDa
70-
55-
40-
100-
70-
55-
40-
35-
25-
70-
55-
B56α
GST
GST-pulldown
Input
B56α
N=3
B56α/ GST Normalized
2.5
2.0
1.5
1.0
0.5
0.0
CIP2A WT
GST
CIP2A WT
GST

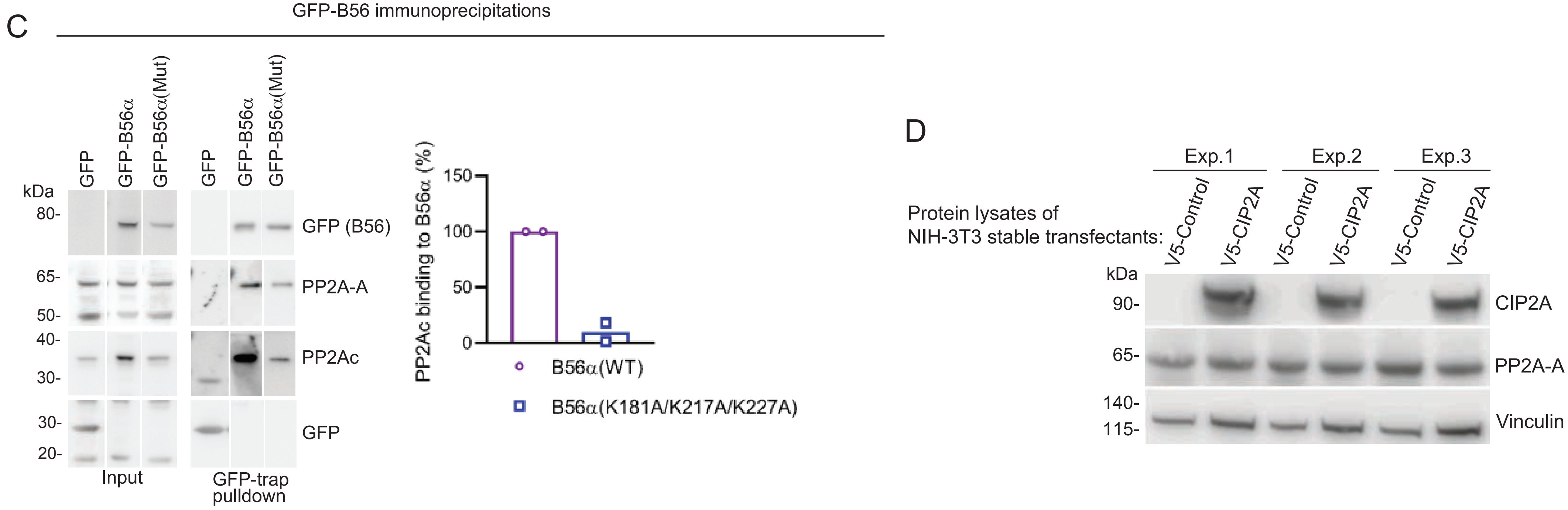

Supplementary Figure 8: (A) Electrostatic charge representation of B56α indicating two regions important for interaction with LxxIxE motif containing substrate proteins: The peptide binding groove (filled with LxxIxE peptide) and acidic patch strengthening the substrate binding to B56α. CIP2A-B56α crosslinks are indicated by black numbers. Both the LxxIxE peptide binding groove and the acidic patch are sandwiched between the CIP2A interaction sites on B56α explaining how CIP2A effectively mutes substrate binding of B56α.

B) GST-pulldown assay of direct protein interaction between GST-CIP2A (1-560) and with wildtype B56a or B56a triple mutant K181A, K217A and K227A. Representative image of three independent biological replicates is shown. Quantification is shwon and mean + S.E.M, N=3.

C) GFP-tagged B56α wild-type (WT) or triple mutant was expressed in HEK293T cells. The amount of bound C subunit to GFP-tagged B56α upon GFP trap was quantified by anti-C immunoblotting. Shown is the mean value of the ratios of the quantified anti-C signal versus the quantified anti-GFP signal, relative to the WT B56α (set at 100% in each experiment), as determined in n= 2 independent experiments. Associated phosphatase activity of B56α, measured on a nonspecific PP2A substrate. Specific PP2A activities were calculated by correcting the measured activities for the pulled B56α, determined by anti-GFP immunoblotting. Results represent the mean value + S.E.M of the pulled activity of B56α WT or triple mutant, relative to the pulled activity of B56α WT (set at 100% in each experiment), as determined in n= 2 (pull-down) or n=3 (PP2A activity) independent biological repeats. A two-sided one-sample t-test (compared with 100%) was used to assess statistical significance for PP2A activity difference (*$p < 0.0277$). D) Western blot analysis of PP2A-A in NIH-3T3 control cells and in CIP2A(1-905) V5 stable cells (B,C,and D) Source data are provided as a Source Data file.
