# Supplementary material for: Structural mechanism for inhibition of PP2A-B56α and oncogenicity by CIP2A
Source: Nat Commun. 2023 Feb 28;14:1143. doi: 10.1038/s41467-023-36693-9 (PMC9974998; doi:10.1038/s41467-023-36693-9)
Supplement: Supplementary file 3 — Description of Additional Supplementary Files [file 41467_2023_36693_MOESM3_ESM.docx]

**Description of Additional Supplementary Files**

File Name: Supplementary data 1

Description: B56 protein interactors

File Name: Supplementary data 2

Description: B56α interactors regulated by CIP2A in AP-MS experiment

File Name: Supplementary data 3

Description: Summary phosphoproteome data from CIP2A K21A clone 1 cells

File Name: Supplementary data 4

Description: Phosphopeptides significantly downregulated in CIP2A K21A clone 1 cells

File Name: Supplementary data 5

Description: Reactome Pathway Analysis of cellular process impacted in CIP2A K21A clone 1 cells
