# Supplementary material for: Structural mechanism for inhibition of PP2A-B56α and oncogenicity by CIP2A
Source: Nat Commun. 2023 Feb 28;14:1143. doi: 10.1038/s41467-023-36693-9 (PMC9974998; doi:10.1038/s41467-023-36693-9)
Supplement: Supplementary file 9 — Reporting Summary [file 41467_2023_36693_MOESM9_ESM.pdf]

nature portfolio

Corresponding author(s): Jukka Westermarck

Last updated by author(s): Jan 24, 2023

Reporting Summary

Nature Portfolio wishes to improve the reproducibility of the work that we publish. This form provides structure for consistency and transparency in reporting. For further information on Nature Portfolio policies, see our Editorial Policies and the Editorial Policy Checklist.

Statistics

For all statistical analyses, confirm that the following items are present in the figure legend, table legend, main text, or Methods section.

n/a	Confirmed	
☐	☒	The exact sample size (*n*) for each experimental group/condition, given as a discrete number and unit of measurement
☒	☐	A statement on whether measurements were taken from distinct samples or whether the same sample was measured repeatedly
☐	☒	The statistical test(s) used AND whether they are one- or two-sided<br>*Only common tests should be described solely by name; describe more complex techniques in the Methods section.*
☒	☐	A description of all covariates tested
☒	☐	A description of any assumptions or corrections, such as tests of normality and adjustment for multiple comparisons
☐	☒	A full description of the statistical parameters including central tendency (e.g. means) or other basic estimates (e.g. regression coefficient) AND variation (e.g. standard deviation) or associated estimates of uncertainty (e.g. confidence intervals)
☐	☒	For null hypothesis testing, the test statistic (e.g. *F*, *t*, *r*) with confidence intervals, effect sizes, degrees of freedom and *P* value noted<br>*Give P values as exact values whenever suitable.*
☒	☐	For Bayesian analysis, information on the choice of priors and Markov chain Monte Carlo settings
☒	☐	For hierarchical and complex designs, identification of the appropriate level for tests and full reporting of outcomes
☒	☐	Estimates of effect sizes (e.g. Cohen's *d*, Pearson's *r*), indicating how they were calculated

Our web collection on statistics for biologists contains articles on many of the points above.

Software and code

Policy information about availability of computer code

Data collection	No software was used for data collection.
Data analysis	For quantification of Western blot images, open source ImageJ-win64 Fiji/ Image J (2.9.0/1.53t) was used. Graphs were plotted using GraphPad Prism (version 6.1 until 9.3.1). CRISPR guides (crRNAs) and the homology repair (HR) template for CIP2A_K21A mutation were designed utilizing the Benchling CRISPR Design tool (www.benchling.com)<br>For XL-MS data analysis, a protein sequence database for the identification of XL peptides was generated by searching the lowest mass SEC fraction for unmodified peptides using Mascot (version 2.5.1, MatrixScience). MS/MS spectra were searched against these databases using xQuest (version 2.1.5 (Walzthoeni et al., 2012). Cross-link identifications were visualized with xiNET (version 1.1) (Combe et al., 2015). MS data in Thermo's proprietary raw format were first converted into the open mzXML format, the format used for xQuest searches described below. Conversion was performed using msconvert.exe, version 3.0.9393, part of the ProteoWizard toolbox, using the following options: --mzXML --32 --filter "peakPicking true 1-2". mzXML files were further converted into mgf format using MzXML2Search, part of the Trans-Proteomic Pipeline (TPP v4.7 rev 0) using default parameters (MzXML2Search –mgf).<br>To identify cross-linked peptide pairs, the mzXML files generated by msconvert were searched against these databases using xQuest (version 2.5.1)<br>For B56 interactome study, peptides were identified by MASCOT (version 2.2.06, Matrix Science). Progenesis LC-MS software (version 4.1.4832.42146, Nonlinear Dynamics) was used for relative uantification of peptides and Proteome Discoverer software (v. 1.4, Thermo Fisher Scientific) was used for peptide validation using the Percolator node.. Qlucore Omics Explorer (version 3.6 was used for statistical data analysis.<br>For phosphoproteomics, protein identification and quantitation were done using Protein Discoverer version 2.5 software (Thermo Scientific Inc. Germany). The MS/MS spectra were deisotoped and deconvoluted by using the MS2 spectrum processor node in Protein Discoverer. Mascot (version 2.7.0) was used as a search engine.<br>MS data was acquired automatically by using Thermo Xcalibur 4.4 software (version 4.4.16.14, Thermo Fisher Scientific). Protein identification and quantitation was performed by processing the raw data from all replicates using Protein Discoverer (PD) version 2.5 software (Thermo

Scientific Inc. Germany) connected to an in-house server running the Mascot 2.7.0 software (Matrix Science). Data was searched against a SwissProt (version 2021_2) database using Homo sapiens taxonomy filter. For phosphosite motif enrichment analysis, motifs were assigned using NetworKIN algorithm (https://networkin.info). The proximity ligation slides were analyzed with laser scanning microscope Nikon Eclipse Ti2-E at 60 × magnification, and images were processed with Fiji-ImageJ (version 2.9.0/1.53t).
Protein structures figures and analysis were generated using Pymol (version 2.3.1). All PDB files used for figures downloaded from the www.rcsb.org database.

For manuscripts utilizing custom algorithms or software that are central to the research but not yet described in published literature, software must be made available to editors and reviewers. We strongly encourage code deposition in a community repository (e.g. GitHub). See the Nature Portfolio guidelines for submitting code & software for further information.

Data

Policy information about availability of data

All manuscripts must include a data availability statement. This statement should provide the following information, where applicable:

- Accession codes, unique identifiers, or web links for publicly available datasets
- A description of any restrictions on data availability
- For clinical datasets or third party data, please ensure that the statement adheres to our policy

All mass spectrometry proteomics data have been deposited to the ProteomeXchange
Consortium via the PRIDE partner repository and are available with following identifiers:
PXD020636 [http://proteomecentral.proteomexchange.org/cgi/GetDataset?ID=PXD020636](XL-MS proteomics data)
PXD030297 [http://proteomecentral.proteomexchange.org/cgi/GetDataset?ID=PXD030297](AP-MS proteomics data)
PXD035179
[http://proteomecentral.proteomexchange.org/cgi/GetDataset?ID=PXD035179](Phosphoproteomics data from K21A mutant cells)

Structural data:
PDB: 5UFL [http://doi.org/10.2210/pdb5UFL/pdb]
PDB: 6NTS [http://doi.org/10.2210/pdb6NTS/pdb]
PDB: 2IAE [http://doi.org/10.2210/pdb2IAE/pdb]

Field-specific reporting

Please select the one below that is the best fit for your research. If you are not sure, read the appropriate sections before making your selection.

- [x] Life sciences
- [ ] Behavioural & social sciences
- [ ] Ecological, evolutionary & environmental sciences

For a reference copy of the document with all sections, see nature.com/documents/nr-reporting-summary-flat.pdf

Life sciences study design

All studies must disclose on these points even when the disclosure is negative.

Sample size	For the orthotopic mammary fat pad xenograft assay, in total of 23 mice were used. Mice were allocated according to body weights to three study groups MDA-MB-231 CIP2A_Control (n=7), MDA-MB-231 CIP2A_K21A Clone1 (n=8) and MDA-MB-231 CIP2A_K21A_Clone2 (n=8), using published algorithm (Laajala, T.D. et al. Optimized design and analysis of preclinical intervention studies in vivo. Scientific reports 6, 30723 (2016).<br>For other experiments the sample size was determined based on similar experiments from previous publications. No statistical method was used to predetermine sample size according to common scientific practices related to the field. In general all the experiments were performed with at least two independent biological repeats.
Data exclusions	For the orthotopic mammary fat pad xenograft assay, among the replicate values, the outliers were identified by Prism 9 using 5 % threshold with ROUT algorithm.<br>For B56 interactome study, only peptides with a q value < 0.01 were considered.<br>For phosphoproteomics, only proteins with q-value < 0.01 (< 1 % FDR) as determined by percolator and detected in both replicates were used for further analysis.
Replication	Number and type of repeats for the experiments is indicated in the figure panels.
Randomization	For the orthotopic mammary fat pad xenograft assay, mice were allocated according to body weights to three study groups MDA-MB-231 CIP2A_Control (n=7), MDA-MB-231 CIP2A_K21A Clone1 (n=8) and MDA-MB-231 CIP2A_K21A_Clone2 (n=8), using published algorithm (Laajala, T.D. et al. Optimized design and analysis of preclinical intervention studies in vivo. Scientific reports 6, 30723 (2016).<br>To our best understanding no randomization was needed for any other experiments based on common scientific practices related to the field.
Blinding	Not used in this study based on common scientific practices related to the field and similar experiments from previous publications (in

addition to this section, for explanation see also "Animals and other organisms" below).

Reporting for specific materials, systems and methods

We require information from authors about some types of materials, experimental systems and methods used in many studies. Here, indicate whether each material, system or method listed is relevant to your study. If you are not sure if a list item applies to your research, read the appropriate section before selecting a response.

Materials & experimental systems

n/a	Involved in the study
☐	☒ Antibodies
☐	☒ Eukaryotic cell lines
☒	☐ Palaeontology and archaeology
☐	☒ Animals and other organisms
☒	☐ Human research participants
☒	☐ Clinical data
☒	☐ Dual use research of concern

Methods

n/a	Involved in the study
☒	☐ ChIP-seq
☒	☐ Flow cytometry
☒	☐ MRI-based neuroimaging

Antibodies

Antibodies used

The following antibodies were used: anti-V5 (monoclonal mouse (mM) Ab (E10/V4RR), Thermo Fisher Scientific, MA5-15253; 1:5,000), anti-V5 (mM, Thermo Fischer Scientific, R960-25; 1:2,000), anti-CIP2A (mM Ab(2G10-3B5), sc-80659, 1:1,000), anti-GST (polyclonal rabbit (pR Ab), Thermo Fisher Scientific CAB4169; 1:10,000), anti-GST (pR Ab, Cell Signaling 2622S; 1:10,000), anti-GST (mM Ab (B-14), sc-138; 1:10,000), anti-PR65 (pR Ab (H-300), sc-15355; 1:1,000 and 1:5,000), anti-B56α (mM Ab (23), sc-136045; 1:500 and 1:5,000), anti-β-Actin (mM Ab (C4), sc-47778; 1:5,000 and 1:10,000), anti-B56γ (mM Ab (E6), sc-374380; 1:500), all from Santa Cruz Biotechnology), anti-c-Myc (mM Ab (9E10), sc-40, 1:1,000), anti-PP2Ac (pR Ab, Cell Signalling 2038S; 1:1,000 and 1:5,000), anti-GAPDH (mM Ab (6C5), Hytest 5G4-6C5 ; 1:10,000), anti-PR65 (clone C5.3D10, 1:1,000) and anti-PP2Ac (clone F2.6A10, 1:500 both generously supplied by Dr. S. Dilworth at Middlesex University, London, UK), anti-GFP (pRb Ab, 2555S, Cell Signaling, 1:1,000), anti-cleaved PARP (mM Ab (E51), ab32064 abcam; 1:1,000), anti-Phospho-MEK1/2 (Ser217/221) (pRb Ab, Cell Signalling 9121S; 1:1,000), anti-phospho-Vimentin (Ser39) (pR Ab, Cell Signalling 13614; 1:1,000), anti-HA tag (mRb Ab (C29F4), Cell Signaling 3724; 1:200 and 1:1,000).

Secondary antibodies used were: polyclonal goat anti-mouse immunoglobulin-HRP (P0447), Dako, polyclonal swine anti-rabbit (P0399), Dako, polyclonal rabbit anti-mouse, P0260, Dako, and anti-rabbit IgG HRP-linked, 7074S, Cell Signaling all used at 1:5,000 dilution. For assays with B56γ and B56α(K181A/K217A/K227A), the following secondary antibodies (IRDye® 680RD Donkey anti-Mouse IgG Secondary Antibody, #926-68072 and IRDye® 800CW Goat anti-Rabbit IgG Secondary Antibody, #926-32211) were used at 1:10,000 dilution.

Validation

All antibodies are validated for species and application by the manufacturer and validation can be found on manufacturers' websites using antibody references provided in the section "Antibodies used": anti-V5 (monoclonal mouse (mM) Ab (E10/V4RR), Thermo Fisher Scientific, MA5-15253, https://www.thermofisher.com/antibody/product/V5-Tag-Antibody-clone-E10-V4RR-Monoclonal/MA5-15253), anti-V5 (mM, Thermo Fischer Scientific, R960-25, https://www.thermofisher.com/antibody/product/V5-Tag-Antibody-Monoclonal/R960-25), anti-CIP2A (mM Ab(2G10-3B5), sc-80659, https://www.scbt.com/de/p/cip2a-antibody-2g10-3b5), anti-GST (polyclonal rabbit (pR Ab), Thermo Fisher Scientific CAB4169, https://www.thermofisher.com/antibody/product/GST-Tag-Antibody-Polyclonal/CAB4169), anti-GST (pR Ab, Cell Signaling 2622S, https://www.cellsignal.de/products/primary-antibodies/gst-antibody/2622), anti-GST (mM Ab (B-14), sc-138, https://www.scbt.com/de/p/gst-antibody-b-14), anti-PR65 (pR Ab (H-300), sc-15355, https://www.scbt.com/p/pp2a-aalpha-beta-antibody-h-300), anti-β-Actin (mM Ab (C4), sc-47778, https://www.scbt.com/p/beta-actin-antibody-c4), anti-B56γ (mM Ab (E6), sc-374380, https://www.scbt.com/p/pp2a-b56-gamma-antibody-e-6), all from Santa Cruz Biotechnology), anti-c-Myc (mM Ab (9E10), sc-40, https://www.scbt.com/p/c-myc-antibody-9e10?requestFrom=search), anti-PP2Ac (pR Ab, Cell Signalling 2038S, https://www.cellsignal.de/products/primary-antibodies/pp2a-c-subunit-antibody/2038), anti-GAPDH (mM Ab (6C5), Hytest 5G4-6C5, https://shop.hytest.fi/product/glyceraldehyde-3-phosphate-dehydrogenase-gapdh-antibody), anti-GFP (pRb Ab, 2555S Cell Signaling, https://www.cellsignal.de/products/primary-antibodies/gfp-antibody/2555), anti-cleaved PARP (mM Ab (E51), ab32064 abcam, https://www.abcam.com/cleaved-parp1-antibody-e51-ab32064.html), anti-Phospho-MEK1/2 (Ser217/221) (pRb Ab, Cell Signalling 9121S, https://www.cellsignal.de/products/primary-antibodies/phospho-mek1-2-ser217-221-antibody/9121), anti-phospho-Vimentin (Ser39) (pR Ab, Cell Signalling 13614, https://www.cellsignal.de/products/primary-antibodies/phospho-vimentin-ser39-antibody/13614), anti-HA tag (mRb Ab (C29F4), Cell Signaling 3724, https://www.cellsignal.de/products/primary-antibodies/ha-tag-c29f4-rabbit-mab/3724).

Secondary antibodies used: IRDye® 680RD Donkey anti-Mouse IgG Secondary Antibody, #926-68072, https://www.licor.com/bio/reagents/irdye-680rd-donkey-anti-mouse-igg-secondary-antibody, IRDye® 800CW Goat anti-Rabbit IgG Secondary Antibody, #926-32211, https://www.licor.com/bio/reagents/irdye-800cw-goat-anti-rabbit-igg-secondary-antibody), polyclonal goat anti-mouse immunoglobulin-HRP (P0447, https://www.agilent.com/en/product/specific-proteins/elisa-kits-accessories/goat-anti-mouse-immunoglobulins-hrp-affinity-isolated-2717109), polyclonal swine anti-rabbit (P0399, https://www.agilent.com/store/productDetail.jsp?catalogId=P039901-2), polyclonal rabbit anti-mouse (P0260, https://www.agilent.com/store/productDetail.jsp?catalogId=P026002-2), all from Dako, and anti-rabbit IgG HRP-linked, 7074S, Cell Signaling. https://www.cellsignal.de/products/secondary-antibodies/anti-rabbit-igg-hrp-linked-antibody/7074.

Eukaryotic cell lines

Policy information about cell lines

Cell line source(s)	The following cell lines were used: 22RV1, MDA-MB-231, HEK-293-T, NIH-3T3; all cell lines used were originally from ATCC.
Authentication	MDA-MB-231 cell line was authenticated by ATCC (01.11.2017) using Short Tandem Repeat (STR) analysis as described in 2012 in ANSI Standard (ASN-0002) Authentication of Human Cell Lines: Standardization of STR Profiling by the ATCC Standards Development Organization (SDO). Other used cell lines were not authenticated. MDA-MB-231 was the only cell line from which we draw any medical or translational conclusions whereas the other cell lines were used only based on their suitability for the given biochemical or cell biology question without any reference to the disease origin of the cell line.
Mycoplasma contamination	Cells were regularly tested for mycoplasma contamination; no contaminated cell lines were used.
Commonly misidentified lines (See ICLAC register)	None.

Animals and other organisms

Policy information about studies involving animals; ARRIVE guidelines recommended for reporting animal research

Laboratory animals	Twenty-three female Athymic Nude mice (Hsd:Athymic Nude-Foxn1nu, Envigo, Gannat, France), weighing between 19-24 g, were used at 6 weeks of age.
Wild animals	The study did not involve wild animals.
Field-collected samples	The study did not involve samples collected from the field.
Ethics oversight	This study has been performed according to the guidelines following the EU legislation related to the use of animals for scientific purposes. National Animal Experiment Board of Finland authorized the animal studies with the license ESAVI/9241/2018 that were performed according to the instructions given by the Institutional Animal Care and Use Committees of the University of Turku.

Note that full information on the approval of the study protocol must also be provided in the manuscript.
